# Variations in Species-Level N:P Stoichiometry of Charophytes and Aquatic Angiosperms on the Tibetan Plateau

**DOI:** 10.3389/fpls.2018.00870

**Published:** 2018-06-22

**Authors:** Zhong Wang, Zhigang Wu, Yang Wang, Dan Yu

**Affiliations:** Department of Ecology, College of Life Sciences, Wuhan University, Wuhan, China

**Keywords:** alpine wetland, aquatic plants, freshwater ecosystems, stoichiometric homeostasis, temperature

## Abstract

The variations in nitrogen (N) and phosphorus (P) stoichiometry between species and along environmental gradients reflects plant growth and survival under certain conditions. Exploring the determinants of plant N and P stoichiometry at species level could help us understand the mechanisms of plant distribution. Temperature is considered a driving factor in forming the geographical patterns of plant N and P stoichiometry at the community level. Here we selected four common aquatic plants to explore the divergence of plant N and P stoichiometry between species and the species-level variations across large geographical gradients on the Tibetan Plateau. We found that plant N and P concentrations and N:P ratios were significantly different among the four species/groups. Charophytes had the lowest N and P concentrations, but the N:P ratio did not differ significantly from those of angiosperms. All four species/groups plant N concentrations were positively correlated with P concentrations. The temperature was also the primary explanatory variable, while the habitats properties showed weak and inconsistent effects on plant N and P stoichiometry. Plant N and P concentrations increased, but N:P ratios decreased, with decreasing temperature. Altitude, rather than latitude, determined the environmental patterns of plant N and P stoichiometry by affecting the temperature. These findings indicated that, after removing the influences of species replacement at the community level, temperature still plays a primary role in forming the geographical patterns of plant N and P stoichiometry at species level. Plants of each species could optimize their investment strategies of elements under different environmental conditions. The Tibetan Plateau is recognized as an area that is sensitive to global warming. Our results provided evidence, in terms of N and P stoichiometry, of potential variations among aquatic plants in nutrient absorption and element cycling under climatic warming.

## Introduction

Aquatic plants are the primary producers in freshwater ecosystems and supply food to the primary consumers (Bornette and Puijalon, 2011). Plants, with their species-specific nutrient concentrations and ratios, determine the amount of nutrients that enter ecosystems, support specific consumers with certain nutrient concentrations, and control the stability of freshwater ecosystems, especially under global change regimes (Brothers et al., 2013). Nitrogen (N) and phosphorus (P) are two vital elements for plant growth and survival (Sterner and Elser, 2002; Ågren, 2008). Biologically, plant nutrient concentrations are species-specific functional traits (Duarte, 1992; Demars and Edwards, 2007). The N and P absorption capacities of plants are under genetic control, and maintain the species-specific tissue concentrations under fluctuating conditions of nutrient supply from substrates, called “stoichiometric homeostasis” (Sterner and Elser, 2002; Elser et al., 2010). This theory explains the inter-species variations in plants under the same environmental conditions. However, plants exhibit relatively greater intra-species stoichiometric variability than heterotrophs (Wang et al., 2012), and show significant variations under different environmental conditions (Reich and Oleksyn, 2004).

At large geographic scale, temperature determines the patterns of plant elemental stoichiometry, by directly controlling plant physiological processes, or indirectly via altering species composition in the local community (Reich and Oleksyn, 2004; He et al., 2008; Zhang et al., 2012; Wang et al., 2015). In cold regions, plants usually invest more N and P to biochemical processes to counterbalance the depressed efficiency of enzymatic reactions, which are restricted by low temperature (Reich and Oleksyn, 2004). Furthermore, low temperature can pick out cold-tolerant species to assemble local community in cold regions (Bornette and Puijalon, 2011), and then enhance the plant N and P concentrations at the community level (Demars and Edwards, 2008; Frost and Hicks, 2012; Xia et al., 2014; Wang et al., 2015). Most previous studies have been carried out at the community level, mixing the direct effects of environmental factors on plant physiological process as well as species replacement (Elser et al., 2010). To differentiate the direct and indirect influences of temperature, stoichiometric studies on widespread species are necessary. The air-water interface can buffer the fluctuations of temperature, causing aquatic plants to be more cosmopolitan than terrestrial plants (Santamaría, 2002). The widespread species of aquatic plants allow us to examine the adaption mechanisms of plants at species level across sufficient environmental gradients.

In this study, we focused on four common aquatic plants species/groups (*Stuckenia filiformis*, *Halerpestes tricuspis*, *Triglochin palustris* and charophytes) on the Tibetan Plateau. *S. filiformis* is one of the most widespread submerged species on the Tibetan Plateau, and forms an underwater community as constructive species (Guo et al., 2010b). Charophytes are macroalgae that are ascribed to green plants Viridiplantae, possess superiority in oligotrophic or brackish waters, and can form dense underwater meadows in favorable conditions (Kufel et al., 2016). *H. tricuspis* and *T. palustris* are two common species inhabiting in riparin zones (Wang and Michio, 2001; Guo et al., 2010a). Climatic change (included warming and the variations in precipitation patterns) in the Tibetan plateau threaten the survival of common aquatic plants. With strong topographic relief and the resulting alpine climatic gradients, the Tibetan Plateau provides an ideal platform to explore the mechanisms for plants adapting to drastically varied abiotic environments. Our aims were to (1) test the inter-specific differences of plant N and P stoichiometry to clarify the effects of taxonomy, and (2) examine the intra-specific variations in plant N and P stoichiometry along environmental gradients, including geographical and climatic variables and habitats properties, to explore the relative importance of the environmental factors at species level.

## Materials and Methods

### Study Area

The field investigation was carried out on the Tibetan Plateau from July to August 2012. The Tibetan Plateau is known as “water tower” in Asia and is the source of many great rivers. Thus, the riparian habitats provide suitable environments for aquatic macrophytes. In addition, 1091 lakes (≥ 1 km^2^ in the area) were recorded in the Tibetan Plateau, and provide another type of aquatic habitat (Wang and Dou, 1998). Furthermore, vast expanses of marshes, numerous ponds and channels provide additional aquatic habitats for plants in the Tibetan Plateau.

Topographically, the elevation of the plateau rises from the southeast to northwest, creating variations in climatic variables and habitats properties. Because of the obstruction of the Himalayas, the warm and wet air current from the Indian Ocean travels to the plateau mainly via the canyon of the Yarlung Zangbo River, which lies in the southeastern part of the Tibetan Plateau. The mean multi-annual precipitation shows decreasing trend from about 800 mm in the southeast to about 20 mm in the northwest of the plateau. The mean air temperatures for the year, January, and July are -5 to 11^°^C, -18 to -6^°^C and 5 to 20^°^C, respectively, which also show a decreasing trend from the southeast to the northwest. On the Tibetan Plateau, the highest temperature is coupled with the greatest water availability on the same period in summer. The growing season on the plateau is from May to September (Wang et al., 2013).

### Species

#### *Stuckenia filiformis* (Persoon) Börner

*Stuckenia filiformis* (Potamogetonaceae) is a perennial, cosmopolitan species and totally submerged in fresh or brackish water. The species mainly occurs on the Tibetan Plateau and adjacent regions in Asia and south and north America. On the Tibetan Plateau, *S. filiformis* is one of the most common species and frequently observed to dominate the aquatic community. The stems of the species are slender and the leaves are linear and sessile. The blossom and fruit period are from July to October (Guo et al., 2010b).

#### *Halerpestes tricuspis* (Maximowicz) Handel-Mazzetti

*Halerpestes tricuspis* (Ranunculaceae) is a perennial and small herb species, and always grows in marshes, wet meadows or spreads to water surface with slender stolons. The species mainly occurs on the Tibetan Plateau and adjacent regions and Mongolia. The basal leaves have a petiole, and the leaf blades are always 3-lobed with an area of less than 3 cm × 3 cm. The florescence last from May to August (Wang and Michio, 2001).

#### *Triglochin palustris* L.

*Triglochin palustris* (Juncaginaceae) is a perennial and slender herb species and always grows in marshes and wet meadows below 4500 m in elevation. The species is cosmopolitan in temperate regions. The basal leaves are linear with the shape of ca. 20 cm in length and ca. 1 mm in width. The blossom and fruit period are from June to October (Guo et al., 2010a).

##### Charophytes

Charophytes are cosmopolitan submerged cryptogams, especially in temperate regions, and prefer calcareous aquatic habitats (Forsberg, 1964; Wiik et al., 2015). Charophyte species have a height of 15–30 cm, and differentiate into rhizoid, stem (axis), and branchlet (Han and Li, 1994). Both stems and branchlets are photosynthetically active.

In this study, we did not identify the species of charophytes but treated all the species as a group and compare their stoichiometric characteristics with those of angiosperms.

### Field Sampling

**FIGURE 1:**
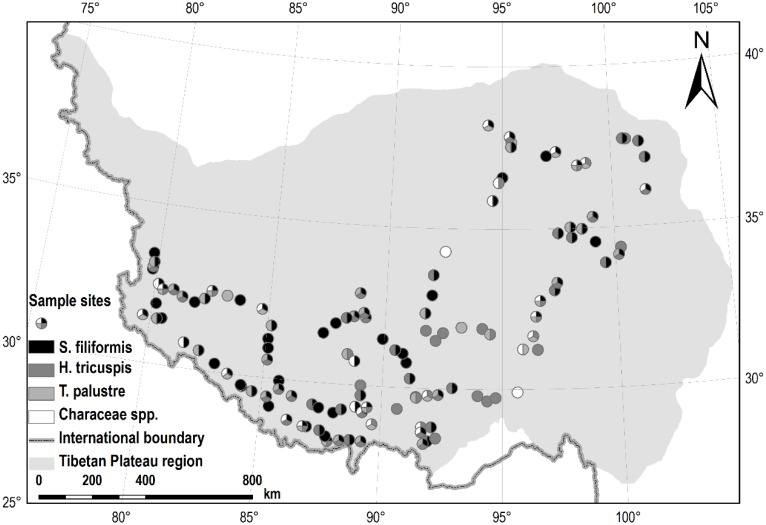
Sample sites in this study. Of all the 126 sites, *S. filiformis*, *H. tricuspis*, *T. palustris* and charophytes were sampled from 98, 73, 54, and 36 sites, respectively.

All of the samples were collected in July and August 2012. For the submerged species, *S. filiformis* and charophytes, 30 segments of plant shoots (ca. 20 cm from the tips) were sampled randomly from each site. All of the leaves (*S. filiformis*) were picked off, while whole shoots samples of charophytes were collected, and put together for each site. For the other two species, *H. tricuspis* and *T. palustris*, we collected 30–50 fully expanded and intact leaves randomly in each site, respectively. All of the samples were oven-dried at 75°C for 48 h, and then finely ground by pulverizer and ball-mill. In total, we investigated 126 sites of aquatic habitats, of which 98, 73, 54 and 36 sites were sampled for *S. filiformis*, *H. tricuspis*, *T. palustris* and charophytes, respectively (**Figure 1**).

For the sediment samples, we dug three vertical and cylindrical cores (20 cm high × 3 cm diameter) randomly in each site. The samples were air-dried and sifted through an 80-mesh sieve. For water samples, we first measured the pH and salinity in the field using a handheld multi-parameter meter (PROPLUS, YSI, United States). A bottle of clean water was collected to measure WTN and WTP.

#### Chemical Measurements

All plants and sediments samples were ground to powder. The C and N concentrations were determined using a CN elemental analyzer (vario MACRO cube, Elementra, Germany), and the P concentrations were measured using the molybdate/stannous chloride method (Kuo, 1996). The N:P ratios were mass-based ratios which were calculated via N concentrations (mg g^-1^) divided by P concentrations (mg g^-1^). For water samples, the WTN and WTP were determined with a photometer (Palintest 7500, Palintest, United Kingdom) within 12 h of collection.

### Data Analysis

The data of plant N and P concentrations and N:P ratios were first log_10_-transformed to normalize their distribution, and the relationships of plant N to P concentrations were regressed by power functions (Reich et al., 2010). Analysis of variance (ANOVA) and Bonferroni-adjusted significance values were employed to assess the differences in plant N and P concentrations and N:P ratios between the species. In this study, we had four groups of data for each variable (N, P and N:P) and performed six times pairwise comparisons in multiple testing of ANOVA. The significance values were adjusted to 0.0083 by dividing 0.05 by the six times of pairwise comparisons. We introduced one climatic variable (GST) and six habitats properties (water pH, salinity, WTN and WTP, and soil total nitrogen and phosphorus) to build general linear models (GLMs) for leaf N and P concentrations and N:P ratios. *F*-tests were used to perform ANOVAs of the GLMs. The percentage of the total sum of squares (%SS) were introduced to quantify the degree of each explanatory variable accounting for in the GLMs (He et al., 2008). In the GLMs, different orders of explanatory variables could not affect the total explaining degree of the model but could vary the degree of each explanatory variable. We entered the explanatory variables in different orders and offered the %SS for each variable when it was the first variable in the GLMs.

Using log_10_-transformed data of plant N and P concentrations and N:P ratios, we applied a family of simple regressions to test the effects of temperature, latitude and altitude on plant N and P concentrations and N:P ratios, respectively. Therefore, each family included 3 sequential tests applying to the same data (e.g., N concentration of each species). The Holm’s Sequential Bonferroni Procedures were introduced to increase the power of the statistical tests (Abdi, 2010). According to the procedure, the original *p*-value was firstly obtained from each test, and then the tests were ordered from the one with the smallest *p*-value to the one with the largest *p*-value. The corrected *p*-value for the *i*th-test, denoted *p*_Bonferroni_, *i*/*C* was computed as:

$$P_{Bonferroni},i/C=(C-i+1)\times p$$

where *C* was the number of tests (3 in this study) and *p* was the original *p*-value of each test. If the original *p*-value of *i*th-test was smaller than the corrected *p*-value, we kept the original one reported in the results. If not, the test was non-significant.

The air temperatures were obtained by entering geographic coordinates into equations derived from data collected at meteorological stations across China. The GST were calculated by averaging the monthly mean temperatures from May to September. All statistical analyses were conducted with R 3.4.2 (R Development Core Team, 2007).

## Results

### Plant N, P Concentrations and N:P Ratios

Results of ANOVA showed that plant N and P concentrations and N:P ratios were significantly different among the four species/groups, and among the three angiosperm species (significance level *p* < 0.001). When pairwise comparisons were performed, the N and P concentrations of charophytes were significantly lower, but the N:P ratio did not differ from their angiosperm counterparts (**Table 1**). Within angiosperms, *S. filiformis* showed the lowest leaf N concentration and leaf N:P ratio, while *H. tricuspis* showed the highest leaf N and P concentrations (**Table 1**).

**Table 1:** Plant N, P concentrations and N:P ratios of the four aquatic species/groups.

Species	*n*	N			P			N:P		
				
		Mean	*SD*	*CV*	Mean	*SD*	*CV*	Mean	*SD*	*CV*
*Stuckenia filiformis*	98	26.13^b^	6.54	0.25	2.97^b^	0.98	0.33	9.51^b^	3.08	0.32
*Halerpestes tricuspis*	73	33.78^a^	7.75	0.23	3.66^a^	1.23	0.34	9.92^ab^	3.15	0.32
*Triglochin palustris*	54	31.13^a^	7.27	0.23	2.85^b^	1.03	0.36	11.83^a^	3.61	0.31
Charophytes	36	10.64^c^	4.92	0.46	1.30^c^	0.85	0.65	9.73^ab^	4.52	0.46


*Different letters indicated significant differences in plant N and P concentrations and N:P ratios between species, at the Bonferroni-adjusted significance level *p* < 0.0083. SD, standard deviation; CV, the coefficient of variation (SD/mean).*

**FIGURE 2:**
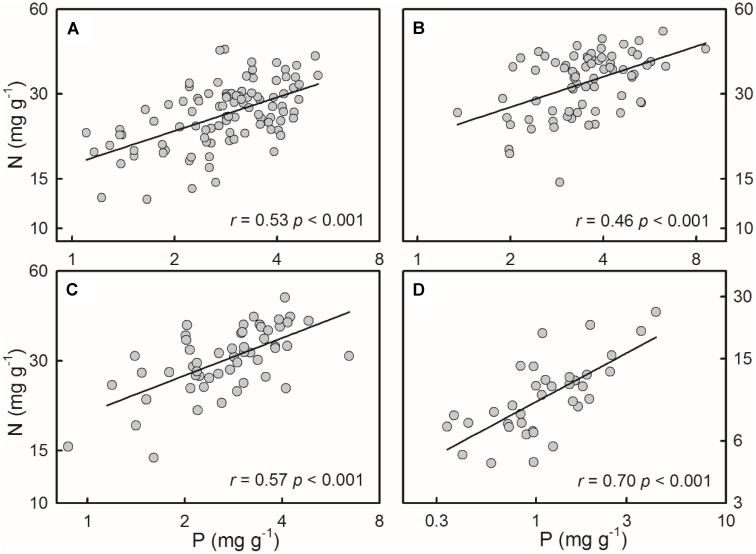
Positive relationships between N and P concentrations of the four species/groups. The correlations were fitted in power functions. **(A)** *S. filiformis*, **(B)** *H. tricuspis*, **(C)** *T. palustris*, and **(D)** Charophytes.

For all of the four species/groups, plant N concentrations positively correlated with P concentrations (**Figure 2**).

### Effects of Environmental Factors on Plant N and P Stoichiometry

The general linear models (GLM) which contained seven explanatory variables explained 16.85 to 42.67%, 20.78 to 47.45%, and 6.75 to 33.87% of the variations in plant N, P concentrations and N:P ratios, respectively (**Table 2**). Among the explanatory variables, GST was the primary variable, while habitat properties showed weak and inconsistent effects on the plant N and P stoichiometry.

**Table 2:** The percentage of the total sum of square (%SS) of each explanatory variable in the general linear model.

Variables	N				P				N:P			
			
	*S. fil*	*H. tri*	*T. pal*	*Cha*	*S. fil*	*H. tri*	*T. pal*	*Cha*	*S. fil*	*H. tri*	*T. pal*	*Cha*
GST	12.79***	18.63***	37.28***	9.16	11.00***	22.03***	41.12***	25.71***	0.87	2.46	9.35*	16.47*
pH	0.86	3.93	0.92	4.28	5.98*	3.64	6.05*	19.02***	4.48*	0.21	5.53	10.51*
Salinity	1.44	0.11	1.11	1.84	0.01	0.30	1.76	10.19*	1.12	0.10	0.65	4.82
WTN	0.05	3.09	1.51	1.72	0.29	3.12	0.11	6.38	0.69	0.23	0.38	4.92
WTP	5.75**	0.95	0.05	0.61	4.56*	0.09	0.10	0.47	0.28	0.23	0.04	3.00
STN	0.34	0.53	3.47	1.44	2.77	6.44*	7.93*	1.15	2.21	4.66	4.16	0.09
STP	0.11	<0.01	1.99	0.01	0.46	0.72	1.34	0.75	0.28	0.82	0.09	1.65
Residuals	74.62	76.91	57.33	83.15	79.22	72.32	52.55	53.69	89.49	93.25	82.11	66.13


*The %SS represents the explanatory degree of each explanatory accounted for. The explanatory variables include growing season mean temperature (GST), water pH value, salinity, water total nitrogen content (WTN), water total phosphorus content (WTP), soil total nitrogen concentration (STN), and soil total phosphorus concentration (STP). *S. fil*, *Stuckenia filiformis*; *H. tri*, *Halerpestes tricuspis*; *T. pal*, *Triglochin palustris*; *Cha*, Charophytes. ^∗^*p* < 0.05, ^∗∗^*p* < 0.01, ^∗∗∗^*p* < 0.001.*

**FIGURE 3:**
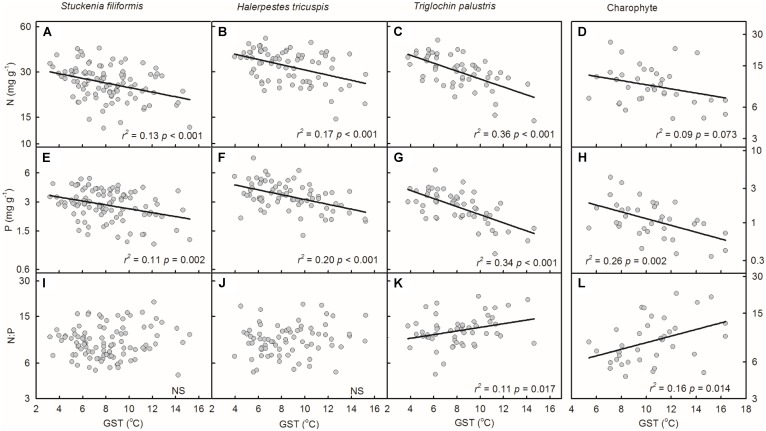
Trends of plant N and P concentrations and N:P ratios along temperature gradients **(A–L)**. The growing season mean temperature (GST) was used to test the effects of temperature on plant N and P concentrations and N:P ratios. The *p*-value in each panel was the original one of each test if it was smaller than the corrected one obtained by Holm’s Sequential Bonferroni Procedure. If not, the test was non-significant (NS).

Plant N and P concentrations of the four species/groups increased with decreasing GST (**Figures 3A–H**). The N:P ratios of *T. palustris* and charophytes decreased with decreasing GST (**Figures 3K,L**), while those of the other two species showed no significant relationships with GST (**Figures 3I,J**).

### Geographic Patterns of Plant N and P Stoichiometry

**FIGURE 4:**
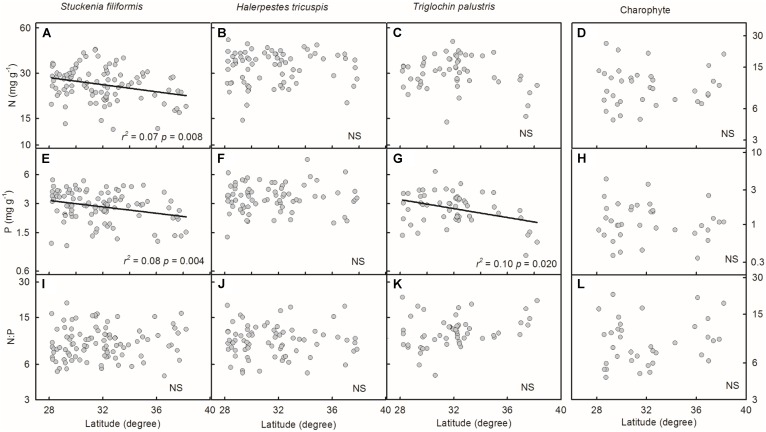
Trends of plant N and P concentrations and N:P ratios along latitudinal gradients **(A–L)**. The *p*-value in each panel was the original one of each test if it was smaller than the corrected one obtained by Holm’s Sequential Bonferroni Procedure. If not, the test was non-significant (NS).

**FIGURE 5:**
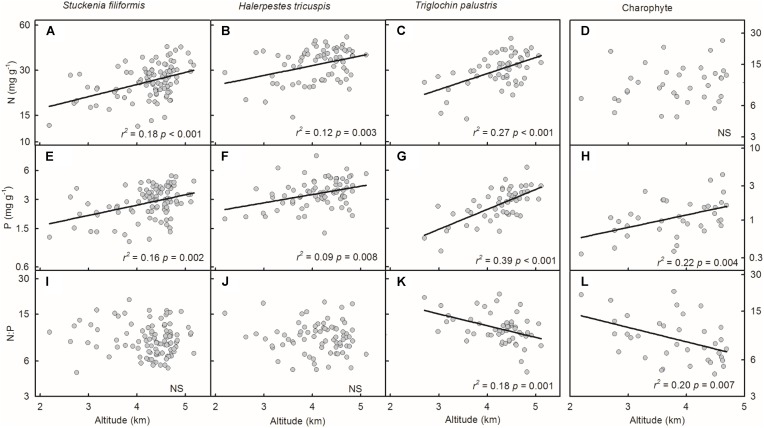
Trends of plant N and P concentrations and N:P ratios along altitudinal gradients **(A–L)**. The *p*-value in each panel was the original one of each test if it was smaller than the corrected one obtained by Holm’s Sequential Bonferroni Procedure. If not, the test was non-significant (NS).

Plant N and P concentrations and N:P ratios of the four species/groups did not show consistent trends along latitude gradients (**Figures 4A–L**). Regarding altitude gradients, plant N and P concentrations of the four species/groups increased toward high altitude (**Figures 5A–H**), except for N concentration of charophytes (**Figure 5D**). The N:P ratio of *T. palustris* and charophytes decreased, but those of the other two species showed no significant relationships with increasing altitude (**Figures 5I–L**).

## Discussion

### Stoichiometric Homeostasis of Aquatic Plants on the Tibetan Plateau

Stoichiometric homeostasis refers that organisms maintain specific element composition by adjusting the organismic physiological processes when faced with variations in nutrient availability in their surroundings (Sterner and Elser, 2002; Elser et al., 2010; Yu et al., 2010). In this study, three patterns were observed. The first was that the plant nutrient concentrations were different among species, especially between angiosperms and charophytes. These results are consistent with Blindow (1992), who stated that charophytes species (*Chara tomentosa* and *Nitellopsis obtusa*) contain less N (10.5 – 14.0 mg g^-1^) and P (0.63 – 0.88 mg g^-1^) than their angiosperm counterparts (N, 24.2 – 24.5 mg g^-1^; P, 2.11 – 2.51 mg g^-1^ in *Myriophyllum spicatum* and *Potamogeton pectinatus*) from shallow lakes in Sweden. Furthermore, several independent studies demonstrated the same patterns when charophytes and angiosperms were taken into account simultaneously (reviewed in Kufel and Kufel, 2002). However, charophytes may not differ so much from angiosperm species as the data shows. Charophytes always build calcite encrustation, which is composed mainly of calcium carbonate, tightly bound to their thalli, and precipitate much more calcium than vascular aquatic plants (Kufel et al., 2016). The encrustation occupies a large proportion (may exceed 70% in extreme situations) of dry plant mass and explains the lower N and P concentrations of charophytes (Blindow, 1992; Kufel and Kufel, 2002; Kufel et al., 2016). In addition, the proportions of encrustation in dry plant mass of charophytes are species-specific (Kufel et al., 2016), inducing a higher value of the coefficient of variation (CV) when charophytes are treated as a group of species, as done in this study (**Table 1**). In further studies, identifying the species, removing the calcite encrustation, and calculating N and P concentrations on an ash-free dry weight basis of charophytes might lead to more accurate conclusions when comparing the nutrient concentrations of charophytes with those of vascular aquatic plants. In the case of the three angiosperm species in this study, mean nutrient concentrations differed from each other at the species levels. As the samples were collected from the same region, such variations were likely attributed to taxonomic differences, rather than the ambient environments (Demars and Edwards, 2007).

Although significant differences observed between the nutrient concentrations of charophytes and their angiosperm counterparts, the N:P ratios of the two plant groups remained within a similar range, and not differ from each other on species mean levels. This was the second principal trend detected in this study. The N:P ratios rather than N and P concentrations are considered to reflect the nutrient limitation of plants (Güsewell and Koerselman, 2002). The similarity of N:P ratios between charophytes and angiosperm species suggests that the lower nutrient concentrations of charophytes did not result from lower ambient nutrient availability since the sampling area for each species were overlapping. On the contrary, innately low nutrient concentrations of charophytes imbue the species with competitive superiority in oligotrophic habitats (Blindow et al., 2014).

The third principal pattern detected here was the positive N-P relationships, which were well documented in previous studies at the species level (Li et al., 2014; Wu et al., 2014) or on community level (Duarte, 1992; Demars and Edwards, 2007; He et al., 2008; Reich et al., 2010). In this study, both charophytes and angiosperms showed coincident positive N-P relationships. Functionally, P is primarily allocated to ribosomal RNA, which is used to synthesize N-rich proteins, indicating that N and P concentrations might co-vary relative to each other to maintain optimal tissue N:P ratios (Sterner and Elser, 2002; Ågren, 2008).

Each species contained specific nutrient concentrations and maintained relatively stable ratios and correlations between elements. The stoichiometric nutrient composition was species-specific, and the plants could control stoichiometric homeostasis in fluctuating environments.

### Stoichiometric Variability of Aquatic Plants and the Effects of the Environment

Empirically, stoichiometric homeostasis is an approximation rather than a strict value, as the organisms always need to respond to the fluctuations of resources availability (Wang et al., 2012). This study revealed that the nutrient concentrations varied considerably within the same species. The N concentrations varied 4-fold and P varied 10-fold within species, indicating the significant influences of the ambient environments. Among all of the environmental factors, temperature played the primary role in determining the patterns of plant N and P concentrations along environmental gradients. Plant N and P concentrations increased with decreasing temperature. Such patterns supported the temperature-plant physiological hypothesis, which suggested that plants invested more N and P in enzyme systems to compensate the depressed efficiency in cold regions (Reich and Oleksyn, 2004). Habitat properties had weak effects on plant N and P stoichiometry (Sardans et al., 2012). Similar results were also reported at the species level for *Ranunculus natans* (Ranunculaceae) in the arid zone of northwest China (Li et al., 2015). Regarding life forms, emergent plants, *T. palustris* and *H. tricuspis*, which leaves were exposed to air directly, had a higher explanatory degree by GST than those of submerged species, *S. filiformis* and charophytes. These results indicate that water buffered the drastic variation of air temperature, and alleviated the stress of low temperature for submerged plants. In all, temperature determined the patterns of plant N and P stoichiometry at the community level (Wang et al., 2015), as well as species level (Li et al., 2015 and this study) across large environmental gradients.

Geographically, temperature decreases with increasing latitude and altitude. Latitudinal patterns of plant element stoichiometry at regional and global scales have been well documented (Reich and Oleksyn, 2004; Han et al., 2005; Xia et al., 2014), whereas altitudinal trends are often neglected. However, altitudinal gradients are known to dramatically alter environmental factors (e.g., temperature) in relatively small areas (Lacoul and Freedman, 2006; Wang et al., 2013; De Long et al., 2015). In this study, the latitude range extended approximately 10 degrees, from 28.18 to 38.21°N, but the altitude range spanned nearly 3000 m, from 2194 to 5176 m, which introduces more drastic variations in temperature than does the latitude range. Leaf N and P had no or weakly significant relationships with latitude, but significantly increased with increasing altitude. The results were consistent with Körner’s (1989) finding that herbaceous plants from high elevation regions contained more nutrients. Therefore, on the Tibetan Plateau, we suggest that the patterns of plant N and P stoichiometry at the species level are determined by the stresses of low temperature, which were induced by altitude rather than latitude.

## Author Contributions

ZWa and DY designed the study. ZWa and ZWu performed the field investigation. ZWa, ZWu, and YW analyzed the data and wrote the manuscript. All authors worked together to produce the final version of the text.

## Conflict of Interest Statement

The authors declare that the research was conducted in the absence of any commercial or financial relationships that could be construed as a potential conflict of interest.

## Abbreviations

GST: growing season mean air temperature
STN: soil total nitrogen concentration
STP: soil total phosphorus concentration
WTN: water total nitrogen content
WTP: water total phosphorus content
